# Supplementary material for: Strongly coupled magneto-exciton condensates in large-angle twisted double bilayer graphene
Source: Nat Commun. 2024 Jun 13;15:5065. doi: 10.1038/s41467-024-49406-7 (PMC11176383; doi:10.1038/s41467-024-49406-7)
Supplement: Supplementary file 1 — Supplementary Information [file 41467_2024_49406_MOESM1_ESM.pdf]

Supplementary Information for

Strongly coupled magneto-excitons in large-angle twisted double bilayer graphene

Qingxin Li *et al.*

Corresponding authors:Xiaoyang Shi, xshi7@albany.edu; Rui Wang, ruiwang@nju.edu.cn;
Geliang Yu, yugeliang@nju.edu.cn; Lei Wang, leiwang@nju.edu.cn

Supplementary Note 1: Decoupled behavior at finite magnetic field.

Here, we analyze the Shubnikov–de Haas (SdH) oscillations at finite magnetic field. Supplementary Fig 2. a shows the plot of longitudinal resistance $\log(R_{xx})$ versus the D and carrier density n at B=4T. Noticeable decoupled behavior, with the same driving mechanism at zero field, is conformed by evolution of different regions(separated by the black dashed lines) in phase diagram. For further analyzing decoupled behavior at finite magnetic field, we present the fan diagram $\log(R_{xx})(n, B)$ at D=1.25V/nm(marked as red rashed line in Supplementary Fig 2. a) as shown in Supplementary Fig 2. b. There are five qualitatively different regions at different n range, which correspond to the different charge carriers occupation in TDBG, which can be interpreted by single-particle band structure shown in Supplementary Fig 2. c-g. In large carrier density like region I and V, both bilayers (top and bottom) are prone to be populated by holes and electrons respectively, illustrated in Supplementary Fig 2. c and g; Reducing carrier density n to region II or IV, only one piece of bilayer(top or bottom) is populated while the other bilayer's fermi energy is in the band gap, illustrated in Supplementary Fig 2. d and f; Finally, in the vicinity of CNP(region III), both bilayers are populated by co-existing hole and electron charge carriers illustrated in Supplementary Fig 2. e. The overlapping electron-hole bands are mark by crossing of red line and blue line, which are on behalf of electron bands and holes bands,respectively.

Supplementary Note 2: Landau levels crossing structure

We here explore the Landau levels crossing structures in the decoupled TDBG model. Taking the spin and valley into consideration, the $N = 0, 1$ Landau levels for τ bilayer, where $\tau = \pm 1$ for the top or bottom bilayer respectively, in the valley ξ are

$$\begin{aligned} E_0 &= \frac{1}{2}\xi u \pm E_Z/2 + \tau D/2 \\ E_1 &= \xi\left(\frac{1}{2}u - \delta\right) \pm E_Z/2 + \tau D/2 \end{aligned} \tag{1}$$

where $\delta = u\hbar\omega_c/\gamma_1$ reflects the valley splitting, and the two layers are distinguished by the on-site energies $\pm\frac{u}{2}$ [1]. D denotes the displacement field. For the higher Landau levels, i.e., $N > 1$, the Landau levels read as

$$E_N^{\pm} = \pm\hbar\omega_c\sqrt{N(N-1)} - \frac{1}{2}\xi\delta \pm E_Z/2 + \tau D/2 \tag{2}$$

When the displacement field varies, the Landau levels of two bilayers tilt in opposite directions, resulting in a crossing of Landau Levels. The lowest two Landau levels i.e. $N = 0, 1$, interlace to form an 8x8 crossing structure shown in maintext Fig. 1e.

Supplementary Note 3: Numerical estimation of the tunneling gap

There are two possible origins for the gap formed between the electron and hole Landau levels in the TDBG. One is the excitonic gap due to the correlation effect, and the other is the hybridization gap due to the electron tunneling between the two bilayers. The second mechanism can be excluded for the case with large twisted angle around 10°. This can be achieved by examining the tunneling strength, i.e., the off-diagonal elements connecting different bilayers of the TDBG Hamiltonian under magnetic fields.

In the Landau gauge, the eigenstate $|N, k_y\rangle$ reads as

$$\psi_{Nk_y}(x, y) = L_y^{-1/2} \exp(ik_y y)\phi_N(x - l_B^2 k_y), \tag{3}$$

with n denoteing the LL index. At zero magnetic field, the TDBG system (valley + or -) can be described by a continuum model:

$$H = \sum_{\mathbf{k}} \Psi_\alpha^\dagger(\mathbf{k}) h_{\alpha,\beta}(\mathbf{k}) \Psi_\beta(\mathbf{k}) + \sum_{\mathbf{k},\mathbf{Q}_j} \Psi_\alpha^\dagger(\mathbf{k}) T_{\alpha,\beta}(\mathbf{Q}_j) \Psi_\beta(\mathbf{k}) \tag{4}$$

where α, β are the combination of layer and sublattice indeices. The second term of Eq.(4) describe the moiré hopping from momentum $\mathbf{k}$(relative to the Dirac point) to momentum $\mathbf{k} + \mathbf{Q}_j$, where $\mathbf{Q}_1 = (0, \frac{4\pi}{3a_M})$, $\mathbf{Q}_2 = (-\frac{2\pi}{\sqrt{3}a_M}, -\frac{2\pi}{3a_M})$ and $\mathbf{Q}_3 = (\frac{2\pi}{\sqrt{3}a_M}, -\frac{2\pi}{3a_M})$. The TDBG under magnetic fields is then described by the Landau quantization of Hamiltonian(4). From Eq.(3) and Eq.(4), we can read off the off-diagonal matrix elements describing the tunneling between the Landau states from different bilayers, i.e.,

$$\begin{aligned} U_{N'k_y',Nk_y} &= w \langle N'k_y'| \exp(i\mathbf{Q} \cdot \mathbf{r}) |N, k_y\rangle \\ &= w\delta_{k_y',k_y+Q_y} \sqrt{\frac{\lambda!}{\Lambda!}} \left(\frac{Q_x + iQ_y}{|\mathbf{Q}|}\right)^{N-N'} \left(\frac{i|\mathbf{Q}|l_B}{\sqrt{2}}\right)^{|N-N'|} \\ &\times \exp\left(-\frac{|\mathbf{Q}|^2 l_B^2}{4} - \frac{i}{2} l_B^2 Q_x (k_y' + k_y)\right) L_\lambda^{|N-N'|}\left(\frac{|\mathbf{Q}|^2 l_B^2}{2}\right) \end{aligned} \tag{5}$$

where λ and Λ denote the cutoff of the Landau level indices N and N', respectively, and L_λ^N is the associated Laguerre polynomial [2, 3].

One observes from Eq.(5) an exponential factor $\exp(-|\mathbf{Q}|^2 l_B^2)$, where $|\mathbf{Q}|$ is given by $4\pi/(3a_M)$ and $a_M = a/(2\sin(\theta/2))$ is the moiré lattice constant. For large twisted angle $\theta \sim 10°$, $|Q|$ is large and the tunneling strength is exponentially suppressed. We take the experimental parameters, i.e., $w = 110$meV, $\theta \sim 10°$, $B = 14$T, and $a = 0.246$nm. Then the hybridization gap, which is of the same order of the tunneling strength, is obtained as $\Delta \sim U \sim 10^{-10}$meV. This is negligible, and is much smaller than the experimentally observed gap Δ_{ex}. Since $\Delta \sim U \sim 10^{-10}\text{meV} \ll \Delta_{ex}$, the interbilayer tunneling can be safely discarded when analyzing the correlation effect, which is dominant and becomes more manifested in the Landau levels physics under magnetic fields.

Supplementary Note 4: Calculation of the excitation gap

To calculate the excitation gap, according to the results in the last section, we discard the negligible tunneling gap and consider two graphene bilayers interacting with each other. The two bilayers are further subjected to a perpendicular magnetic field, thus exhibit Landau quantization characterized by the Landau index N_1, N_2 respectively. The excitonic gap is only favored in the balanced case where the electron and hole densities are equal, thus we focus on the case $N_1 = N_2 = N$, i.e., the electron and hole states are filled to the same Landau level. This leads to a twofold pseudospin degeneracy of the studied system.

The kinetic energy is completely suppressed under strong magnetic fields with well formed Landau levels. We thus need to project the Coulomb interaction onto the Landau levels. The interactions before projection reads as,

$$H_C = \frac{1}{2}\int d^2\mathbf{x} d^2\mathbf{y} V_{\alpha,\beta}(\mathbf{x}-\mathbf{y})[\rho_\alpha(\mathbf{x}) - \rho_{\alpha,0}][\rho_\beta(\mathbf{y}) - \rho_{\beta,0}], \tag{6}$$

where $\rho_{\alpha,0}$ is the average electron density which is determined by $\rho_{\alpha,0} = \nu_\alpha/2\pi l_B^2$, and the indices α, β are the pseudospin indices denoting the two bilayers. $V_{\alpha,\beta}(\mathbf{x})$ is the Coulomb potential and it has the following Fourier components

$$V_{tt}(\mathbf{q}) = V_{bb}(\mathbf{q}) = \frac{2\pi}{|\mathbf{q}|}, \tag{7}$$

$$V_{tb}(\mathbf{q}) = V_{bt}(\mathbf{q}) = \frac{2\pi}{|\mathbf{q}|} e^{-d|\mathbf{q}|}, \tag{8}$$

where d is the spacing between the two bilayers which is set to $d/l_B = 0.1$ according to the experiments. The layer spacing within each bilayer is insignificant compared to d and is

neglected here. The next step is to project Eq.(6) onto the subspace of each Landau level, which leads to

$$V_N^{\alpha,\beta}(\mathbf{q}) = V_{\alpha,\beta}(\mathbf{q})F_N^2(q) \tag{9}$$

where $F_N(\mathbf{q}) = L_n(l_B^2 q^2/2)\exp(-l_B^2 q^2/4)$ is the Landau level form factor, and $L_N(x)$ is an Laguerre polynomial [4].

In the absence of a displacement field, the top and bottom bilayers are symmetric in terms of their band structures. Under magnetic fields, and electron and hole Landau level can exhibits the same energy, leading to the crossing point as shown by Fig.2c of the main text. Interestingly, the correlation effect can lift the degeneracy of the LLs at the crossing points. For example, the attractive Coulomb force between an electron and a hole then would favor formation of excitons, leading to excitonic gap after condensation. Besides, the effect of the intra-bilayer interaction V_{tt} and V_{bb} also need to be carefully analyzed.

To analyze the correlation physics at the LL crossing points, we evaluate the excitation gaps with fully taking into account the interactions among the LLs. To this end, it is more convenient to introduce the Landau site basis [4], in which the Hamiltonian in Eq.(6) is cast into:

$$H_C = \sum_{mnij} V_{mnij}^{\alpha,\beta}\left[\rho(n,m)^{\alpha} - \nu^{\alpha}\delta_{m,n}\right]\left[\rho(j,i)^{\beta} - \nu^{\beta}\delta_{i,j}\right], \tag{10}$$

and

$$V_{mnij}^{\alpha,\beta} = \frac{1}{4\pi}\int d^2\mathbf{k} V_N^{\alpha,\beta}(\mathbf{k})\left\langle m\right| e^{i\mathbf{X}\mathbf{k}}\left|n\right\rangle\left\langle i\right| e^{-i\mathbf{X}\mathbf{k}}\left|j\right\rangle, \tag{11}$$

where $V_N^{\alpha,\beta}(\mathbf{k})$ is the projected coulomb interaction which is dependent on LLs, and $\rho^{\alpha}(n,m)$ is the Landau site density operator, where n, m denotes the degenerate states within each Landau level N.

We firstly calculate the energy cost to excite a particle-hole pair on top of the filled Landau state. For $N_1 = N_2 = N$, both bilayers have the $N > 0$ LLs occupied, and we assume that Landau levels $0, 1, 2, \ldots, N-1$ are completely filled. Then, one considers the electron-hole pair state given by

$$\left|e_k; h_l\right\rangle = c_t^{\dagger}(k)c_b(l)\left|g\right\rangle, \tag{12}$$

where the ground state has the form $\left|g\right\rangle = \Pi_n c_b^{\dagger}(n)\left|0\right\rangle$, and $\left|0\right\rangle$ denotes the vacuum state.

The excitation gap corresponding to the particle-hole excitation is then evaluated via

$$\begin{aligned}\Delta E &= \langle e_k; h_l| H_C |e_k; h_l\rangle - \langle g| H_C |g\rangle \\ &\approx \frac{1}{4\pi}\int d^2\mathbf{k}(V_{tt}(k)+V_{bb}(k))\left[L_N(\frac{l_B^2k^2}{2})\right]^2 e^{-\frac{l_B^2k^2}{2}}.\end{aligned} \tag{13}$$

This result is clearly LL dependent. The dependence of gap on the field B is then obtained as $\Delta E(B) \sim 1/l_B \sim \sqrt{B}$, as a direct result of the Landau form factor.

Certainly, it's crucial to recognize that, owing to the distinct electron and hole environments, their interactions differ when dealing with charged excitations. In this situation, electrons experience stronger screening than holes. We use the parameter 'w' to quantify the strength of screening effects. We model the electron side Coulomb interaction as $2\pi e^{-w_e q l_B}/|\mathbf{q}|$, while the hole side potential is $2\pi e^{-w_h q l_B}/|\mathbf{q}|$, here the parameter w_e and w_h quantify the strength of screening effects of electron and hole sides. Since the screening effect of carriers is weak when filling the zero-energy Landau level starting from hole side, we take $w_h = 0.1$. Conversely, the screening effect is already significant when carriers fill the electron side, so we take the average screening effect for electron side as $w_e = 1.2$. Hence, similar to the calculation of Eq.(13), taking into account the variation in screening parameter, our calculations reveal that the energy of particle-hole type low-energy excitation on the electron side is lower than that on the hole side (shown in the Supplementary Fig 9)

In addition to particle-hole excitations, there is another typical charged excitations, i.e., the skyrmion and anti-skyrmion excitations. Treating the bilayer degrees of freedom as pseudospins, the excitations can carry nontrivial pseudospin textures. In our system where $d/l_B \neq 0$, the $\hat{z}$ component of the order paramgeter is massive and the system has U(1) symmetry. In this scenario, topologically stable charged objects called meron and antimeron form, which have opposite vorticity, but carry the same topological charge($\pm$ $e/2$) and can pair to skyrmion or antiskyrmion which both carry unit of topological charge($\pm$ e) but with opposite charge. The skyrmion or anti-skyrmion configuration in the pseudospin textures are energetically favorable[5], which should be compared in energetics with the electron-hole excitations. Such pseudospin structures bear topological nontrivial properties, which was investigated in the context of $SU(M)$ quantum Hall ferromagnets. The minimum excitation energy for a Skyrmion pair is $\Delta_{SK} = 8\pi\rho_s$ [6, 7], where

$$\rho_S = \frac{1}{32\pi^2}\int_0^\infty q^3 V_N^{eff}(q)dq \tag{14}$$

denotes the stiffness of the order parameter and V_N^{eff} is the projected Coulomb potential[6, 7]. As discussed above, the tunneling between the two bilayers is negligible for $\theta \sim 10^{\circ}$, and thus the TDBG can be treated as an untwisted double bilayers when calculating the skyrmion pair excitation energy. In this scenario, the effective model here is described by the $SU(M) \times SU(M)$ quantum Hall ferromagnets with $M = 4$. Importantly, one observes that the stiffness in Eq.(14) is independent of M. Thus, from Eq.(14), the skyrmion pair excitation energy Δ_{SK} is obtained as $\Delta_{SK} \sim e^2/l_B \sim \sqrt{B}$, where l_B is the magnetic length.

Both of the particle-hole and the spin-texture excitation energys are related to stiffness ($\rho_s \sim 1/l_B$), consequently, the excitation energy is proportional to the Coulomb energy ($E_c = e^2/l_B$). We show the comparison between two types excitation gap as a function of the LL index n on both of hole and electron sides leads to Supplementary Fig 9(FIG. 3d of the main text).

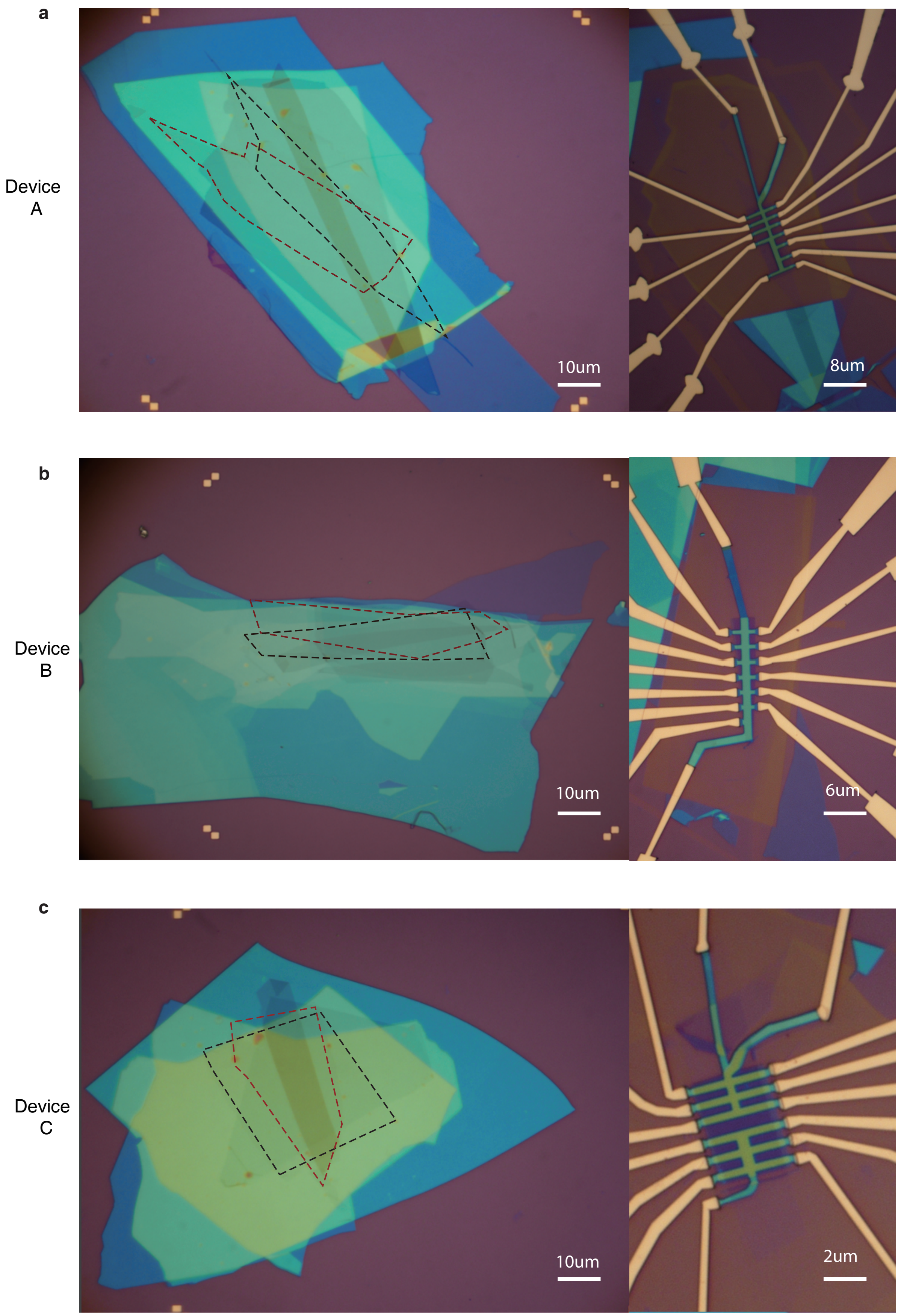

Supplementary Fig. 1. **Optical image of devices.** Optical image of device A in **(a)**, device B in **(b)**, device C in **(c)**. All devices are near 10°. The dashed red and black lines denote two bilayer graphene sheets.

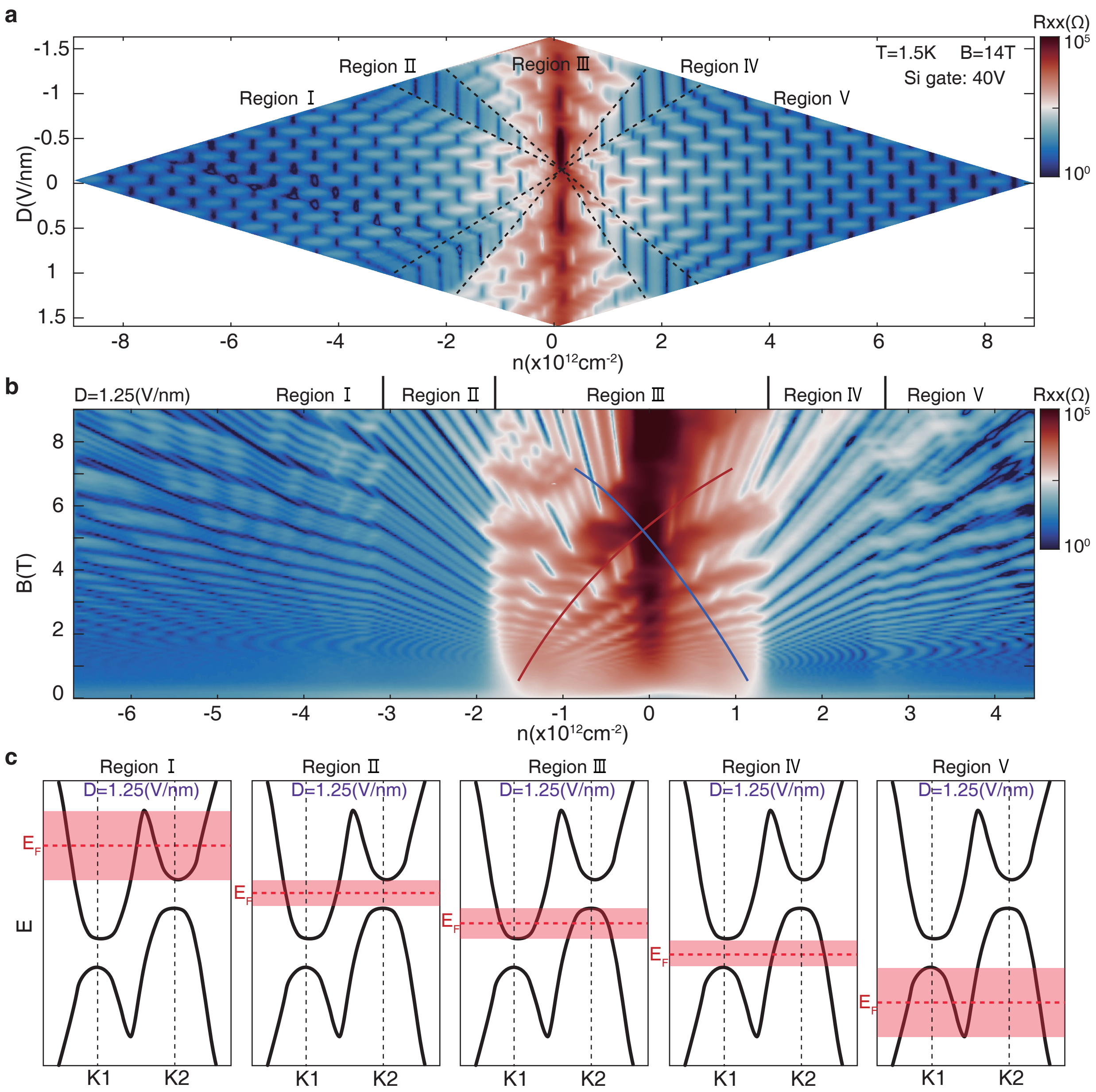

Supplementary Fig. 2. **Decoupled behavior at finite magnetic field. a,** Longitudinal resistance R_{xx} of TDBG at B=4T and T=1.5K, versus n and D. Black lines separate different regions which correspond to different charge carriers polarization in two bilayers. **b,** Longitudinal resistance R_{xx} versus B and n at D=1.25V/nm and T=1.5K. Red and black lines in region III marked electron bands and holes bands, respectively. **c,** Schematic of the band structure near K point. $K1$ and $K2$ represent the K point of bottom and top bilayer, respectively. Red shaded areas represent the energy range of corresponding regions.

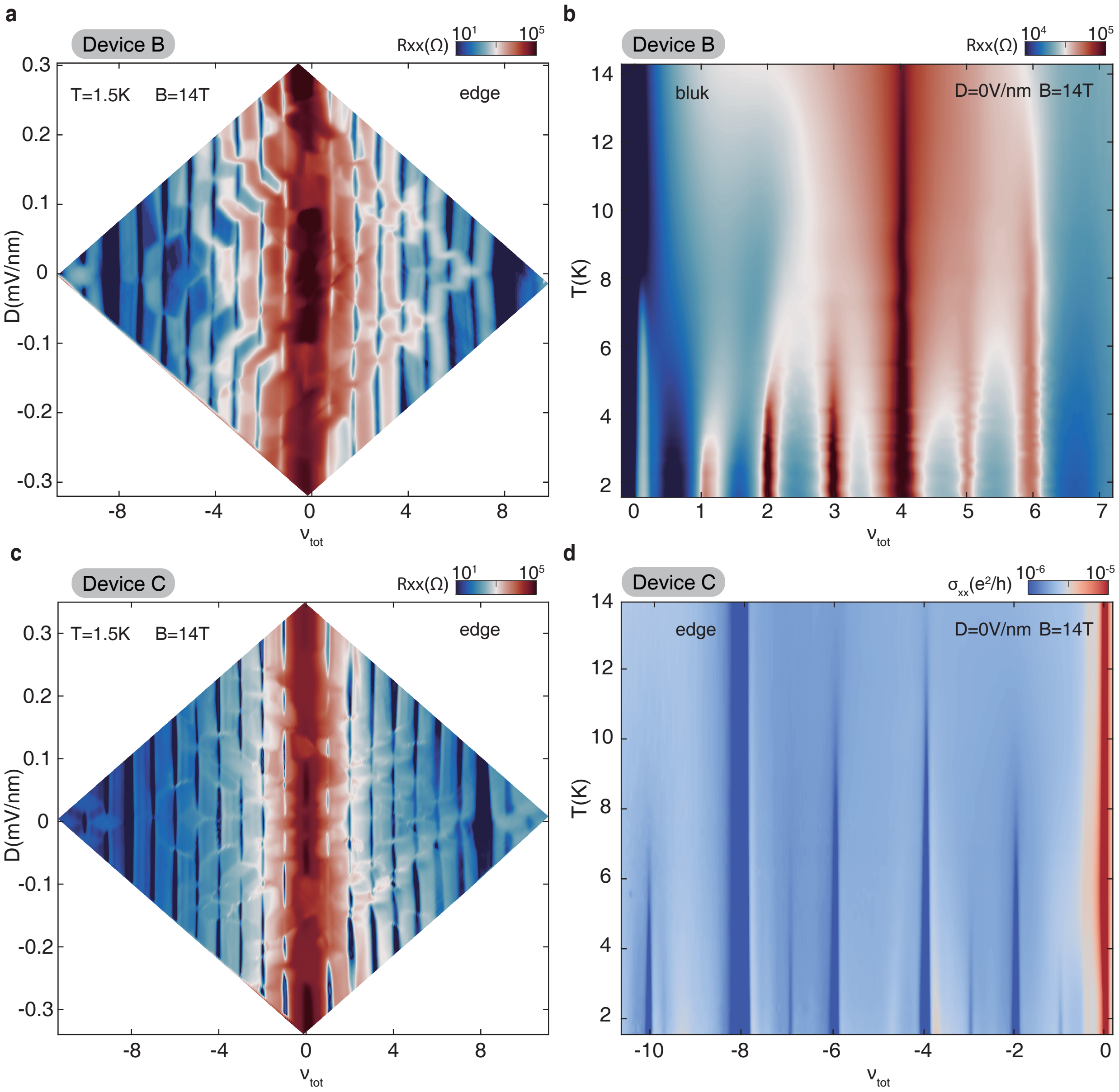

Supplementary Fig. 3. **the 8X8 structure and temperature dependence of ECs in other devices. a-c,** Longitudinal resistance R_{xx} of TDBG at $B = 14$ T and $T = 1.5$ K, versus ν_{tot} and D in device A(a) and device B(c). **b,** Bulk resistance $R_{xx-bulk}$ versus T and ν_{tot} at D=0V/nm in device A. **d,** Longitudinal conductance σ_{xx} versus T and ν_{tot} at D=0V/nm in device B.

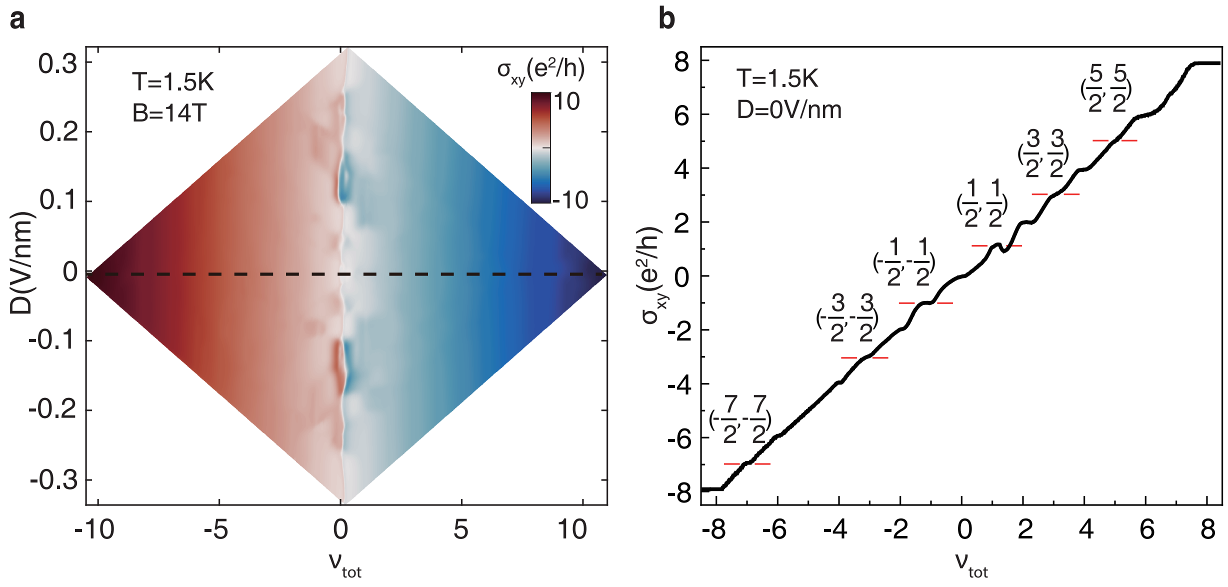

Supplementary Fig. 4. σ_{xy} **of all ECs. a,** σ_{xy} versus D and ν_{tot} at $B = 14$ T and $T = 1.5$ K. **b,** The linecut of (a) along the black dashed line at $D = 0$ V/nm. A series of anomalous states at $\nu_{tot} = -7, -3, -1, 1, 3$ and 5 are marked by red crosses with quantized Hall conductivity and accompanying vanishing longitudinal conductivity(see maintext Fig. 2a). Given that the tunneling is negligible here, this phenomenon implies the emergence of a correlation energy gap due to many-body interactions. When both bilayers are half-filled, interlayer interactions prompt electrons in one bilayer and holes in the other to form magneto-excitons and condense into an incompressible superfluid: exciton condensate.

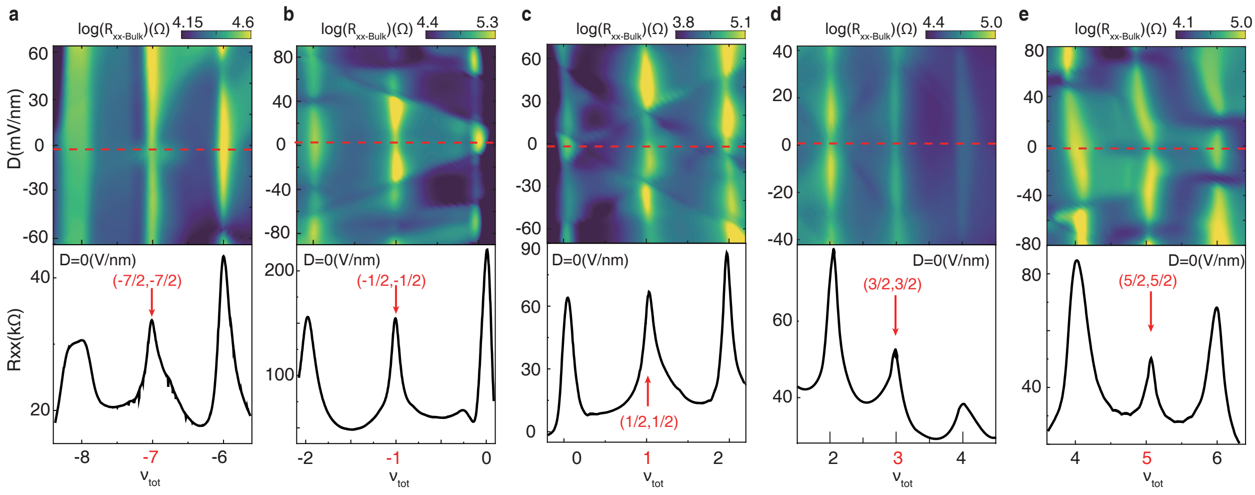

Supplementary Fig. 5. **All ECs at** $D = \mathbf{0V/nm}$. **a-e,** Bulk resistance $R_{xx-bulk}$ versus total filling factor ν_{tot} and displacement field D at $B = 14$ T around $D = 0$V/nm for ν_{tot}=-7, -1, 1, 3, 5. Each bottom panel is a linecut along the grey dashed line in the top panel, which tracks the equal topmost LL population in the two layers.

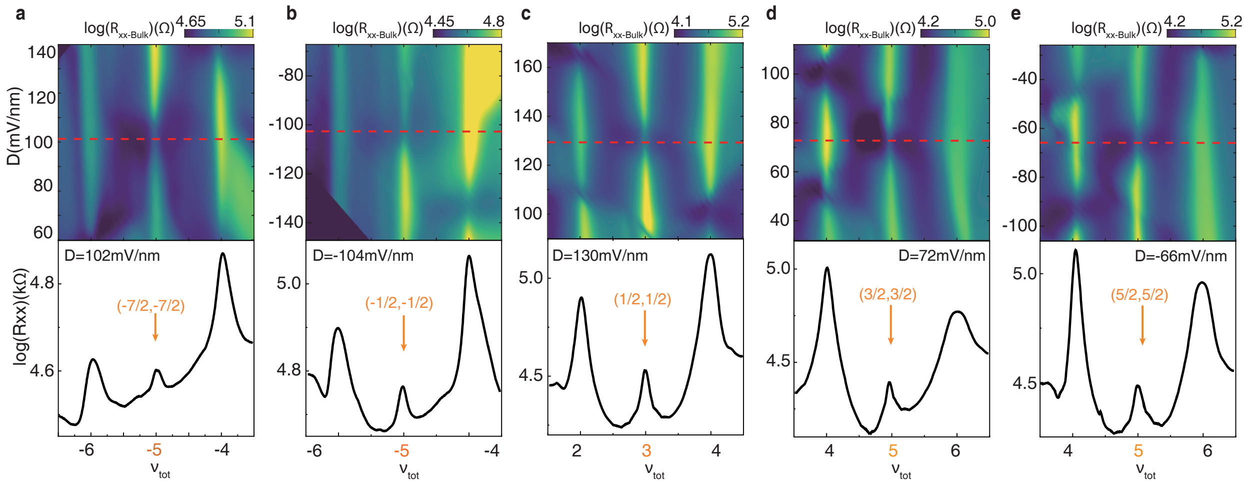

Supplementary Fig. 6. **All ECs at** $D \neq \mathbf{0}$. **a-e,** Bulk resistance $R_{xx-bulk}$ versus total filling factor ν_{tot} and displacement field D at $B = 14$ T when $D \neq 0$. Each bottom panel is a linecut along the grey dashed line in the top panel, which tracks the equal topmost LL population in the two layers.

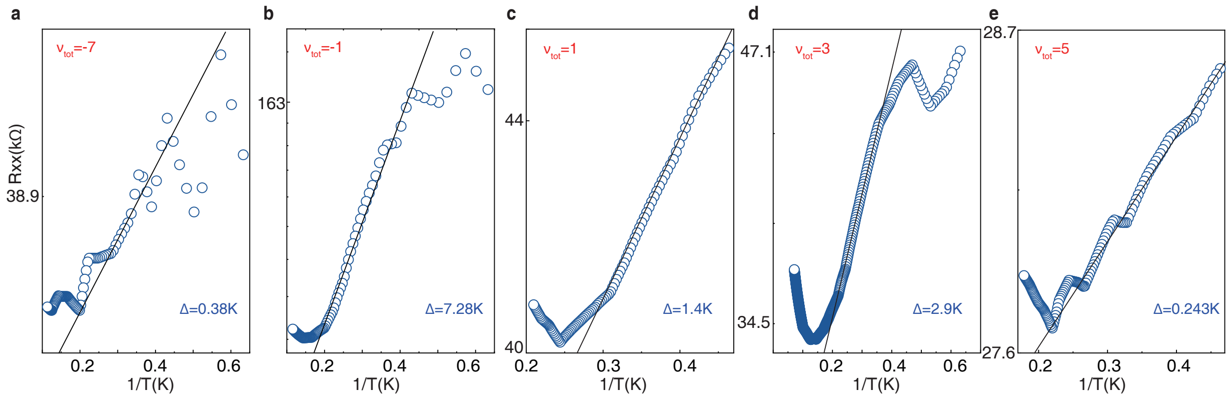

Supplementary Fig. 7. **Arrhenius Fits for all ECs at ν_{tot} =-7, -1, 1, 3, 5. a-e,** Logarithm of bulk resistance $\ln(R_{xx})$ versus $1/T$ at D =0V/nm at ν_{tot} =-7, -1, 1, 3, 5.

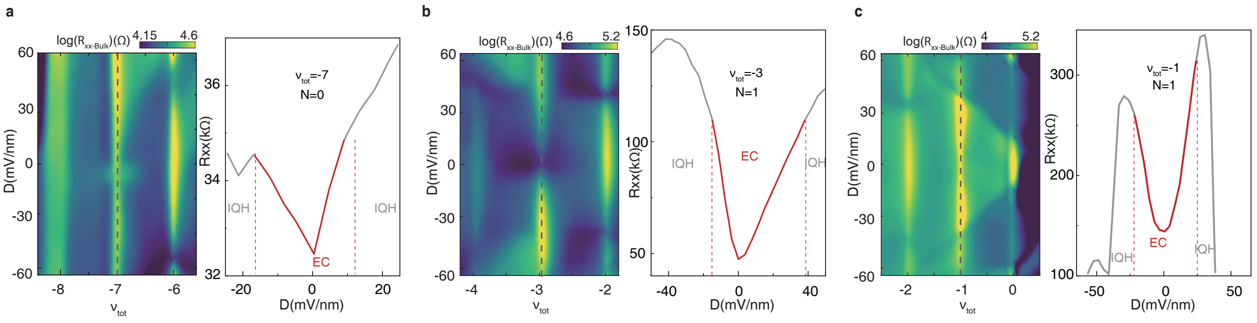

Supplementary Fig. 8. **Layer imbalance of ECs on hole side. a-c,** $R_{xx-Bulk}$ as a function of D for the ECs at ν_{tot}= -7, -3, -1 which correspond to N= 0, 1 and 1 LL, respectively. Red curve regions mark ECs and grey curves correspond to IQH regime.

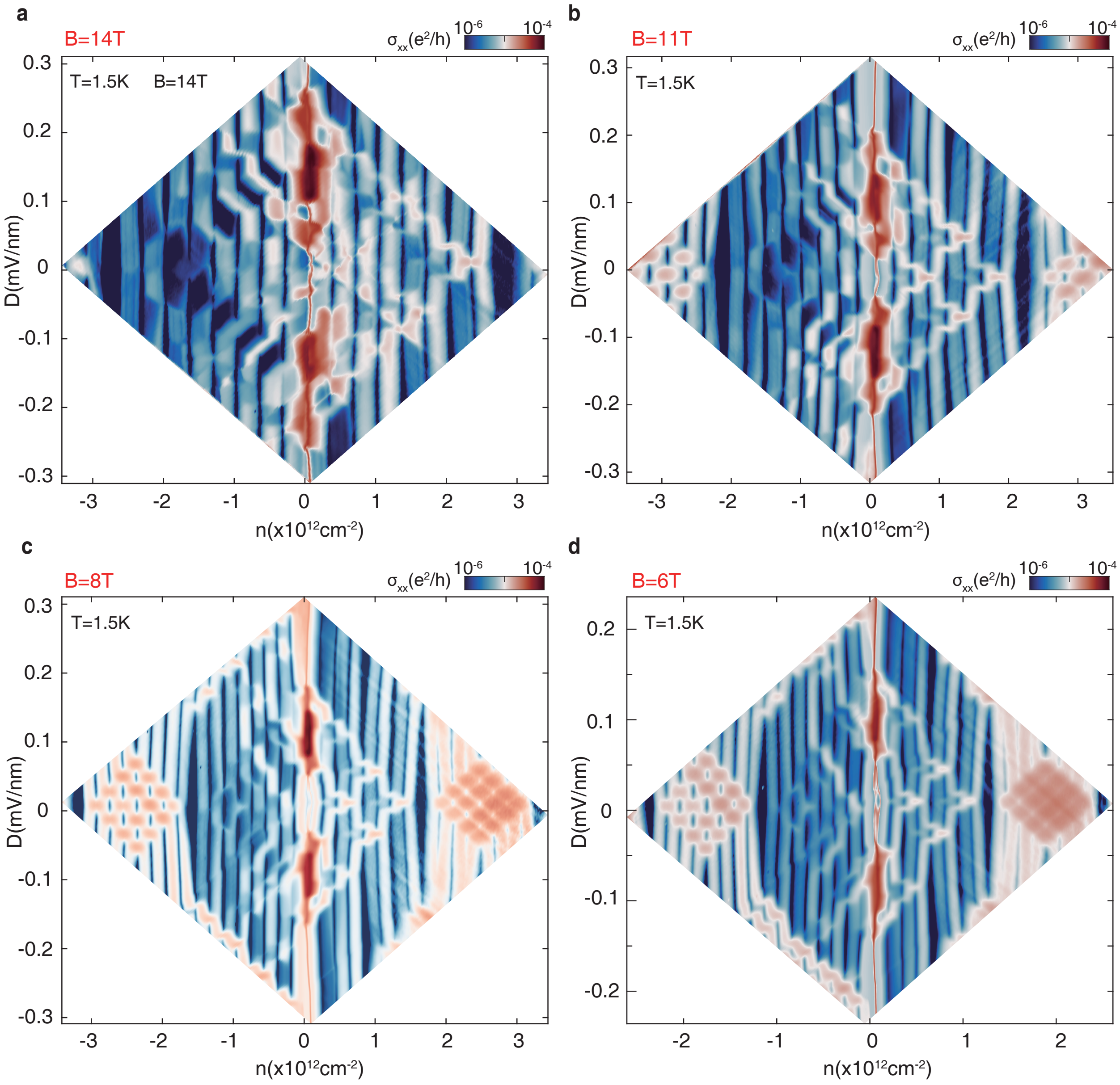

Supplementary Fig. 9. **ECs in the '8X8' matrix at different magnetic fields a-d,** The longitudinal conductivity σ_{xx} versus D and ν_{tot} at $T = 1.5$ K and $B = 14$ T, 11 T, 8 T, 6 T. when $B = 14$ T, ECs appearing at $\nu_{tot} = -7, -3, -1, 1, 3$ and 5 manifest as vanishing longitudinal conductivity states, and as the magnetic field weakens, these ECs gradually diminish, giving way to finite conductivity in these fillings.

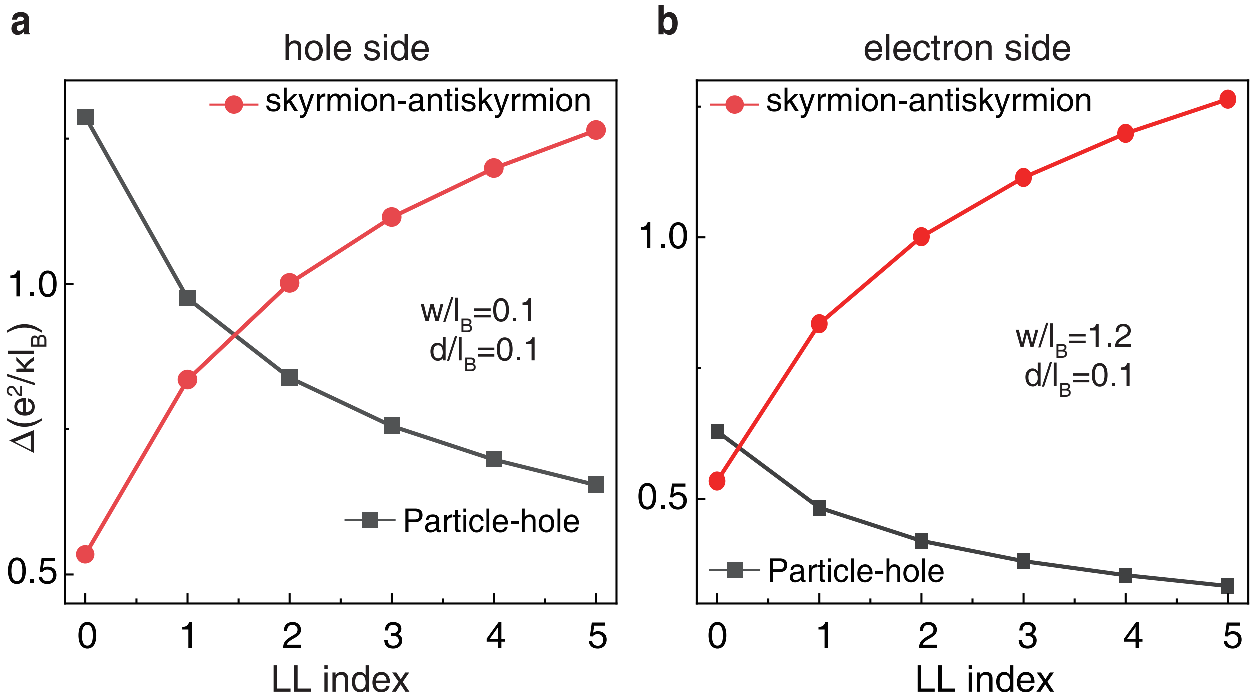

Supplementary Fig. 10. **The comparison between two types excitation gap with Landau level index on electron and hole sides. a-b,** Theoretical calculations of energy gap for two types of excitations on hole and electron sides, at $d/l_B = 0.1$ and $D = 0$ V/nm. The spin-texture excitation energy increases with LL index while particle–hole excitation energy decreases with LL index. Their crossing points for hole and electron sides appear at different positions of LL index due to the screening strength increasing with total filling. The filled markers represent the lowest-energy excitations of ECs.

Supplementary References

[1] E. McCann and V. I. Fal'ko, Physical review letters **96**, 086805 (2006).

[2] D. Pfannkuche and R. R. Gerhardts, Phys. Rev. B **46**, 12606 (1992).

[3] J. A. Crosse, N. Nakatsuji, M. Koshino, and P. Moon, Phys. Rev. B **102**, 035421 (2020).

[4] Z. Ezawa and Z. Ezawa, *Quantum Hall Effects: Field Theoretical Approach and Related Topics* (World Scientific, 2000).

[5] K. Moon, H. Mori, K. Yang, S. M. Girvin, A. H. MacDonald, L. Zheng, D. Yoshioka, and S.-C. Zhang, Phys. Rev. B **51**, 5138 (1995).

[6] K. Yang, S. Das Sarma, and A. H. MacDonald, Phys. Rev. B **74**, 075423 (2006).

[7] D. P. Arovas, A. Karlhede, and D. Lilliehöök, Phys. Rev. B **59**, 13147 (1999).
